# Chemical signalling within the rumen microbiome

**DOI:** 10.5713/ab.23.0374

**Published:** 2023-12-29

**Authors:** Katie Lawther, Fernanda Godoy Santos, Linda B Oyama, Sharon A Huws

**Affiliations:** 1 School of Biological Sciences/Institute for Global Food Security, Queen’s University Belfast, Belfast, BT9 5DL, UK

**Keywords:** *N*-acyl-homoserine lactone (AHL), Autoinducer-2 (AI-2), Autoinducers, Ecology, Gene Regulation, Quorum Sensing

## Abstract

Ruminants possess a specialized four-compartment forestomach, consisting of the reticulum, rumen, omasum, and abomasum. The rumen, the primary fermentative chamber, harbours a dynamic ecosystem comprising bacteria, protozoa, fungi, archaea, and bacteriophages. These microorganisms engage in diverse ecological interactions within the rumen microbiome, primarily benefiting the host animal by deriving energy from plant material breakdown. These interactions encompass symbiosis, such as mutualism and commensalism, as well as parasitism, predation, and competition. These ecological interactions are dependent on many factors, including the production of diverse molecules, such as those involved in quorum sensing (QS). QS is a density-dependent signalling mechanism involving the release of autoinducer (AIs) compounds, when cell density increases AIs bind to receptors causing the altered expression of certain genes. These AIs are classified as mainly being *N*-acyl-homoserine lactones (AHL; commonly used by Gram-negative bacteria) or autoinducer-2 based systems (AI-2; used by Gram-positive and Gram-negative bacteria); although other less common AI systems exist. Most of our understanding of QS at a gene-level comes from pure culture *in vitro* studies using bacterial pathogens, with much being unknown on a commensal bacterial and ecosystem level, especially in the context of the rumen microbiome. A small number of studies have explored QS in the rumen using ‘omic’ technologies, revealing a prevalence of AI-2 QS systems among rumen bacteria. Nevertheless, the implications of these signalling systems on gene regulation, rumen ecology, and ruminant characteristics are largely uncharted territory. Metatranscriptome data tracking the colonization of perennial ryegrass by rumen microbes suggest that these chemicals may influence transitions in bacterial diversity during colonization. The likelihood of undiscovered chemicals within the rumen microbial arsenal is high, with the identified chemicals representing only the tip of the iceberg. A comprehensive grasp of rumen microbial chemical signalling is crucial for addressing the challenges of food security and climate targets.

## RUMEN MICROBIOME

The rumen, the main fermentative compartment of the ruminant forestomach, hosts a complex and dynamic microbial population composed of bacteria, protozoa, fungi, archaea and bacteriophages [1,2]. The rumen microbial ecosystem is characterised by its high cell density, which plays a crucial role in the efficient breakdown of ingested plant material and the overall digestive processes of ruminant animals; mainly characterised as symbiosis [3]. The rumen ecosystem is also versatile, with much redundancy (overlap of function among multiple species) and resilience (resistance to, and capacity to recover from perturbation) observed [3]. The regulation and balance of ruminal fermentation are influenced by various factors, including nutrient balance and a wide range of ecological interactions among microbial populations such as, predation, competition, mutualism, commensalism, and parasitism and amensalism [3–7]. Within this highly complex rumen microbiome, there has been evidence of multiple mechanisms driving the ecological interactions, including use of chemical signalling. However, chemical signalling data in the rumen microbiome is scarce. In this review we focus on outlining the main quorum sensing (QS) systems currently known within the rumen context.

## MICROBIAL CHEMICAL SIGNALLING – QUORUM SENSING

Based on pure bacterial culture studies, QS has been shown to be intrinsically linked to altered gene expression, through the intracellular synthesis of autoinducers (AIs) before their subsequent release and accumulation in the nearby external environment. Environmental concentrations of these AIs increase alongside increases in cell density, until they reach a detection threshold and begin binding to receptors, triggering transduction cascades and leading to the transcription of specific genes [8,9]. QS based systems have also been shown as being intrinsically important in biofilm formation and thereafter in the dispersion of bacteria from mature biofilms [10]. To date, there have been five types of discovered QS systems based on the AIs used, summarised as N-acyl-homoserine lactones (AHLs), autoinducer-2 (AI-2), autoinducing peptides (AIPs), methyl dodecenoic acid, and quinolones [11].

**Figure 1:**
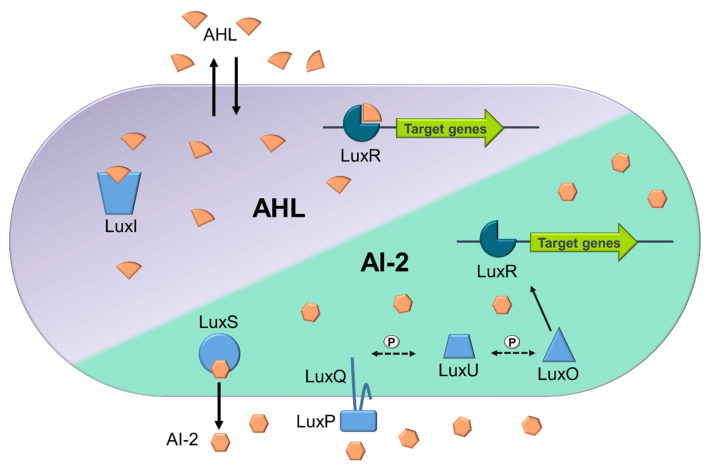
Simplified overview of AHL and AI-2 based quorum sensing mechanisms in *Vibrio harveyi*. The LuxR receptors are in dark blue, while other QS enzymes (LuxI, LuxS, LuxQ, LuxP, LuxU, and LuxO) are in light blue. Phosphate transfers in the signalling cascade are indicated by ‘P’ on dashed arrows, and black arrows show the movement and interaction of molecules. AHL QS: Produced by LuxI, AHL binds to LuxR at high concentrations, inducing target gene expression. AI-2 QS: Generated by LuxS, AI-2 interacts with the LuxPQ complex (LuxP as the periplasmic binding protein and LuxQ as the sensor kinase) at high concentrations. This triggers a dephosphorylation cascade, with LuxU as an intermediary, leading to the inactivation of LuxO, the repressor of LuxR. Inactivated LuxO allows LuxR to activate favouring the induction of target gene expression.

Among those, AHLs are one of the most widely studied QS AI molecules, used mainly by Gram-negative bacterial species, possessing variations in carbon length and compound structure [9,12]. AHL-mediated QS was originally identified in *Vibrio fischeri* (*Aliivibrio fischeri*), best known as the LuxI/LuxR regulatory system. This two-component system comprises of the *luxI* and *luxR* genes, encoding the LuxI protein (which functions as the AHL synthase) and LuxR protein (acting as a signal receptor) respectively [12]. Briefly, AHLs are synthesised by the LuxI synthase, through upregulation of the *luxI* gene, the AHLs then diffuse into the environment. As the concentration of AHL increases in the local environment, more will diffuse into the cytoplasm of nearby cells, binding to the LuxR receptor, triggering the expression of select genes and behavioural changes (Figure 1; [13]). This QS system is thought to only enable intraspecies communication due to AHL molecules only being detected by species that produce that same type of AI [9]. In addition to AHL based QS, *Vibrio harveyi* can also utilise AI-2 based QS and are therefore capable of employing multiple QS systems [14]. Differently to the AHL based QS, the AI-2 based QS enable ‘universal’ interspecies communication and gene regulation [8]. In this QS system, the AI-2 based QS, is facilitated by LuxS (AI-2 synthase and exporter), encoded by the *luxS* gene. Many homologues of this gene have now been discovered in both Gram-negative and Gram-positive bacteria (Figure 1). Once AI-2 signalling molecules are exported, and reach a threshold in the extracellular environment, they will bind to the LuxPQ complex. This then begins a phosphate transfer and the signal is transduced where LuxU is dephosphorylated leading to dephosphorylation of LuxO, resulting in increased LuxR concentrations and altered gene expression (Figure 1; [14]). Gram-positive bacteria, like *Staphylococcus aureus*, can also utilise short macrocyclic peptides known as AIPs [15]. These self-inducing AIPs can regulate gene receptors, often following one of the two common types of circuits known as Agr-like and RNPP-like [15]. Briefly, in Agr-like circuits the peptide signal is not physically transported, unlike in the RNPP-like circuits where the peptide signal will be physically imported before activating the transcription receptor [15,16].

## QUORUM SENSING IN THE RUMEN

Many reviews are available focusing on QS in pathogenic bacteria [e.g 17–20], however, this review introduces the concept of QS with a more system studies based approach, thereby enabling an in-depth discussion of QS systems in the rumen as outlined below.

The majority of studies to date regarding QS in the rumen have focused on laboratory pure cultures of bacteria, using *in vitro* assays and reporter strains for detection of AIs. These *in vitro* methods, including culture-dependent approaches, allow us to gain fundamental knowledge on how rumen bacteria utilise QS under tightly controlled and reproducible conditions [21]. Additionally, recent studies have demonstrated evidence of QS and potential impact on the microbiome by mining rumen bacterial genomes, metagenomes and metatranscriptomes [6,22–25].

### AHL-based quorum sensing in the rumen

Culture-dependent *in vitro* methods have been employed to investigate AHL-based QS in rumen isolate bacteria [26,27]. Erickson et al [26] used two AHL reporter systems, *Chromobacterium violaceum* CV026 and *Agrobacterium tumefaciens* (*Agrobacterium radiobacter*) A136 (pCF372)(pCF272) to detect AHLs. Although AHLs were detected in ruminal fluid samples, no AHLs were observed in the following pure rumen isolates cultures: *Anaerovibrio lipolyticus* 5S, *Fibrobacter succinogenes* S85, *Megasphaera elsdenii* LC1, *Prevotella albensis* 223/M2/7, *Prevotella brevis* GA33, *Prevotella bryantii* B14, *Prevotella ruminicola* 23, *Prevotella ruminicola* 85, *Ruminobacter amylophilus* 70, and WP109, *Selenomonas ruminantium* HD4, four more unnamed *S. ruminantium* strains, and *Succinivibrio dextrinosolvens* 24, as well as several strains of *Butyrivibrio fibrisolvens* (which stains Gram-negative but has a Gram-positive ultrastructure) [26]. The presence of AHL based QS activity in rumen fluid, but not in pure cultures tested, led the authors to hypothesise that either these pure culture bacteria were simply not AHL producers, or that QS only occurs in the density-dependent manner in mixed culture rumen microbiomes [26]. However, there has since been evidence that bacteria isolated from the rumen can produce AHLs in *in vitro* conditions, for example in *Pseudomonas aeruginosa* [27]. When *Escherichia coli* was transformed with the *luxI* gene homolog identified in the rumen isolated *P. aeruginosa*, changes in expressions were observed for the following genes *fliC*, *gadA* and *sdiA* [27]. These genes are known to be linked to motility, increased acid tolerance and QS respectively [28].

To date the findings from culture based and *in vitro* rumen fluid-based experiments remain highly variable. The methodologies employed can explain some of this variability, including experimental alterations such as primer differences, limitations of the bioassays for screening QS molecules and the difficulty of emulating the highly competitive rumen environment *in vitro*. For example, within the cattle gastrointestinal tract (GIT), current evidence suggests that AHL-based QS only occurs in the rumen, with no evidence of QS in other sites of the GIT [29]. However, limitations in our current QS molecule detection technologies could explain this apparent absence of AHLs in the lower GIT. Another potential reason for AHL absence, could be that the AHL-producing microbes are simply lacking in this area but are present in the rumen microbiome. An alternative reason could be due to the pH differences between the lower GIT and the rumen. The pH of the acidic rumen is the ideal environment where the homoserine ring of AHLs are stable, in comparison the alkaline pH of the lower GIT can lead to the hydrolysis of this ring, degrading the QS molecule and negating the effectiveness of the signal being generated [29]. The pH of the rumen can be driven by diet, with diet formulation including grain to roughage ratios having a great impact on pH and inappropriate diets leading to nutritional disorders in cattle such as ruminal acidosis [30]. Despite Erickson et al [26] finding that animals receiving concentrate diets tended to have longer chain AHLs in their rumen fluid in comparison to those receiving forage diets, overall AHL synthesis in the rumen was found not to be diet dependent and the impact of diet on QS in the rumen is yet to be clarified.

To date evidence of QS have been reported in the cattle ruminal fluid samples but not in the caprine ruminal fluid samples [29,31]. When the rumen fluid of Liuyang black goats was investigated, no evidence of QS, including AHLs and AI-2 activity, was identified [31]. In this study, the primary method utilised to identify AHLs was gas chromatography-mass spectrometric (GC-MS) and the authors highlighted potential problems with this method notably the short retention times of the AHLs standards, which potentially led to the lack of AHLs detected. This lack of QS evidence in this caprine rumen study could also be due to several reasons, for example that the rumen of goats did not harbour organisms that are capable of QS such as those seen in cattle [31]. In contrast, when considering other established molecular based methods, homologues of *luxS* found in *P. ruminicola* were identified both *in vitro* and *in vivo* from goat rumen fluid indicating that the rumen microbiomes of goats do have the potential ability to communicate through AI-2 QS [31] and that the detection method could play a crucial part in the detection of AHLs in the caprine rumen and should be considered further.

The variability of results in the above-mentioned experi ments highlights the need to employ both culture-dependent and culture-independent methods for investigating QS in the rumen. Indeed, numerous studies have now utilised genomic, metagenomic, or metatranscriptomic analysis, revealing a large diversity of potential QS mechanisms within the rumen [6,23,24]. For example, the advent of the Hungate collection, which sequenced 410 rumen bacteria and 21 archaea isolate cultures, allowed the mining of these genomes against known QS genes [23,32]. Won et al [23] found evidence of AHL QS, although not widespread, with only one species of Gram-negative bacteria, *Citrobacter* sp. NLAE-zl-C269, possessing an AHL synthase gene. Interestingly, this *Citrobacter* species genome also contained *luxS* and *luxR* genes representing the potential to engage in multiple QS systems, i.e., both AHL and AI-2 [23]. Liu et al [24] expanded this mining of rumen genomes to include 948 bacterial genomes and 33 archaeal genomes, sourced from Shi et al [33], Gharechahi et al [34], and GenBank [35]. In this study they found more extensive evidence of AHL QS, with 5 archaeal genomes (all from the genus *Methanobrevibacter*) and 58 bacterial genomes containing AHL genes, mainly harboured by the following orders; Eubacteriales, Bacteroidia, Clostridiales, and Selenomonadales [24].

### AI-2 based quorum sensing in the rumen

In contrast to AHL based QS, evidence suggests AI-2 QS is far more widespread within the rumen. Through the utilisation of the *V. harveyi* BB170 bioluminescence assay, *B. fibrisolvens*, *Eubacterium ruminantium*, *Ruminococcus flavefaciens*, and *Succinimonas amylolytica* have been confirmed as producing AI-2 like molecules [36]. These findings, indicating that *R. flavefaciens* species are capable of AI-2 production, are further supported through genome sequencing of these isolates, confirming that they harbor *luxS* gene homologues [36]. In contrast, no *luxS* gene homologues were identified from the other 3 species that were identified as being capable of QS AI-2. Interestingly, *luxS* genes have been identified in *P. ruminicola*, yet this species did not produce AI-2 molecules under the experimental conditions employed by Mitsumori et al [36]. Further investigations on *Prevotella* species by Gorenc et al [37], utilising an optimised *V. harveyi* BB170 autoinducer bioassay, reported evidence of AI-2 type QS by *P. ruminicola*-like strain 223/M2/7A. Contrary to this, *P. bryantii* strains were unable to induce such a bioluminescence assay response, suggesting a lack of AI-2 QS system in this strain [37]. Additionally, the predominant rumen bacterium, *Streptococcus bovis*, has been identified as harbouring the QS *luxS* gene, through the use of *S. bovis* genomic DNA as a PCR template and degenerate primers for the *luxS* gene [38]. Through molecular techniques, including northern blot analysis, *S. bovis* was also found to be capable of transcribing the *luxS* gene, however the production of the LuxS protein was not found to be directly related to cell density [38]. In contrast, Mitsumori et al [36], did not detect the *luxS* gene in the genome of *S. bovis*. It should be noted that such differences in findings could potentially be due to primer or culturing differences or indeed strain differences [38].

Beyond pure culture experiments, there is evidence of AI-2 QS in rumen fluid using culture independent techniques [22–24]. Ghali et al [22] mined 3 bovine rumen metagenomic and one metatranscriptomic datasets for the presence of *luxS* genes, with 135 *luxS* genes identified predominantly from the phyla Bacteroidota (genus *Prevotella* mainly) and Firmicutes (*Ruminococcus albus* mainly), and to a lesser extent Fusobacteria and Actinomycetota. Further to this, through metatranscriptomic analysis, 34 *luxS* were expressed largely by Bacteroidetes, Firmicutes and Spirochaetes [22]. Another study, Won et al [23], prospecting the Hungate collection [32], found AI-2 lux-based genes in 191 bacterial genomes, across both Gram positive and negative, 139 and 53 genomes respectively, and were most abundant in *Butyrivibrio*, *Prevotella*, *Ruminococcus*, and *Pseudobutyrivibrio* genera, all of which are prevalent in the rumen. In the same study, metatranscriptomic datasets were prospected and showed that LuxS proteins were the most widespread followed by LuxR, while the LuxS synthase was highly expressed by *Prevotella* species [23]. Similarly, AI-2 genes were identified as highly expressed using metatranscriptomic based methods by Liu et al [24]. This study also detected that AI-2 genes are widespread across rumen bacterial genomes, with 680 out of the 948 ruminal bacteria genomes mined containing AI-2 genes, including the following genera of bacteria; *Prevotella*, *Butyrivibrio*, *Ruminococcus*, *Oribacterium*, *Selenomonas*, and *Treponema* genera [24].

### Other mechanisms of quorum sensing in the rumen

In addition to AI-2 and AHL mediated communication, other methods of QS have been identified in pure cultures of ruminant bacteria, however, research into these mechanisms is still in its infancy. For example, *S. gallolyticus* subsp. *gallolyticus* has been shown to have the ability to produce peptide pheromone competence-stimulating peptides, or CSP, involved in the ComABCDE QS pathway [39]. The peptide variant produced by *S. gallolyticus* subsp. *gallolyticus* differs from those previously seen in others *Streptococcus* spp. The CSP produced by *S. gallolyticus* subsp. *gallolyticus* is not thought to be involved in competence induction (as previously demonstrated by other *S. gallolyticus* subspecies) but instead plays a role in QS and regulation of bacteriocin-like inhibitory substances, suggesting that this QS system leads to a competitive advantage through the ability to eliminate close competitor species [39]. Additionally, autoinducing peptide, AIP, based QS genes have been identified in the genomes of ruminal bacteria, however evidence is limited [24]. AIP based QS genes, *agrA/agrB*, were identified in 199 rumen bacterial genomes, mainly all belonging to the Firmicutes phylum [24]. These genes are part of the Agr operon, *agrB*, *agrD*, *agrC*, and *agrA*, which together form a quorum-sensing circuit most widely studied in *Staphylococci* species [40]. This AgrBDCA QS system, requires the combination of AgrB and AgrB to process and secret AIPs, the two component signal transduction system, AgrAC, then responds to the AIPs [41,42]. This AIP detection then leads to the up-regulation of RNAIII, the agr effector molecule, which initiates regulatory changes in virulence gene expression in bacteria such as *Streptococci* spp. [40]. AIP QS has also been identified in Firmicutes (Bacillota), a dominant taxon in the rumen, in other cellulolytic environments [43]. Namely landfill leachate (liquid that passes through landfills and in the process extracts soluble or suspended solids, or any other component of the landfill), where uncultured clostridial species (*Clostridium thermocellum*-like [*Acetivibrio thermocellus*]) was identified as harbouring the *agrC* AIP gene [43].

## CONSEQUENCES OF QUORUM SENSING IN THE RUMEN

Although there is now a range of evidence that QS is prevalent within the rumen microbiome there is still a limitation in our understanding of how QS influences gene regulation within this environment and consequently its impacts on rumen ecology and the host phenotype. However, limited data does exist that suggests QS can influence rumen colonisation [6,29,44]. For example, QS may play a key role in enterohemorrhagic *E. coli* (EHEC) cattle GIT colonisation. As the natural asymptomatic reservoir, the cattle GIT can be successfully colonised by EHEC, and this colonisation will result in the cattle shedding the pathogen into the environment. However, EHEC does not have its own *luxI* gene nor can it produce AHLs, instead relying on the AHLs produced by other bacteria, another indicator of interspecies QS mechanisms [45]. This interspecies QS by EHEC is enabled through the LuxR receptor homologue, SdiA, a transcription factor capable of sensing and reacting to environmental AHLs [28]. The AHLs present in rumen fluid can regulate the expression of genes associated with commensal EHEC colonisation ensuring the pathogen can successfully pass through the rumen and reach the desired colonisation site, the recto-anal junction [29,44,46]. AHLs can repress transcription of genes through SdiA, including the locus for enterocyte effacement gene cluster which are genes required for attaching and effacing (A/E) lesions, preventing EHEC colonisation in the hostile rumen environment. While SdiA simultaneously activates expressions of others such as *gad* genes, which allows for increased acid survival, enabling passage through the stomach [44–46].

**Figure 2:**
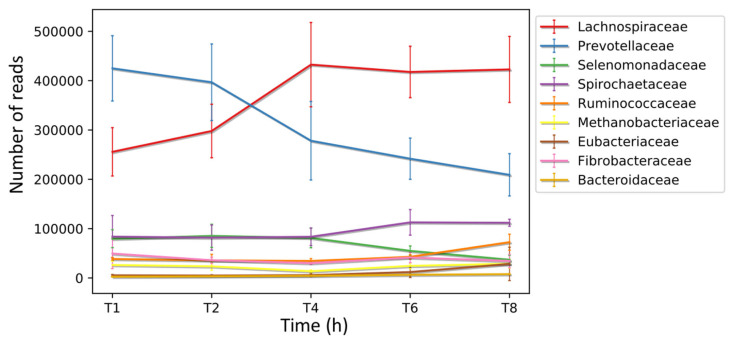
Biphasic colonization of fresh perennial ryegrass in the rumen. Putative coding sequences (CDS) were predicted in the *de novo* assembled transcriptomes obtained from the biofilm interface of fresh perennial ryegrass using an *in sacco* incubation approach. Subsequently, these CDS were taxonomically analysed at different time points during colonization, and the number of reads assigned to each taxon is presented at the family level., analysis completed by Huws et al [6] where error bars represent standard deviations.

Furthermore, multiple studies investigating the temporal colonisation of fresh perennial ryegrass (PRG) in the rumen, including the ability to form biofilms, have shown that colonisation is biphasic with similarities in attached communities from 0 to 4 h (primary colonisation) and thereafter from 4 to 24 h (secondary colonisation) a change in the attached community occurs [6,47–49; Figure 2). Likewise, such colonisation within the rumen is dominated by the biofilm phenotype [6,47–49]. The biofilm phenotype is intrinsically linked to QS, with QS known to be an essential a factor in biofilm formation, maturation and dispersion [10,50].

**Figure 3:**
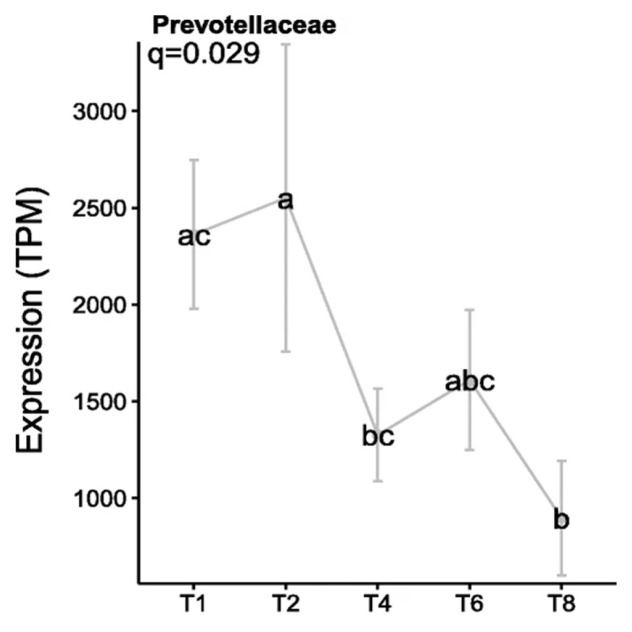
Expression levels of the *luxS* gene by *Prevotellaceae* colonising fresh perennial ryegrass (transcripts per million, TPM). Using an *in sacco* incubation approach the biofilm interface of fresh perennial ryegrass was characterized by metatranscriptomic profiling over different incubation times. The incubation times (h) are indicated on the X axis, error bars represent standard deviation, while different letters (abc) indicate significance between time points, (Huws et al [6]; Figure 8).

The factors which cause this change from the primary to secondary sub microbiomes were recently investigated using metatranscriptomics, with the resultant data showing that AHL was not identified as a QS mechanism in the PRG-colonising bacteria. In contrast, AI-2 QS appeared to be more abundant, which is perhaps unsurprising as this aligns with previous findings based on *in vitro* and genomic technologies [6,23,24]. In particular, the *luxS* gene was expressed abundantly by *Prevotellaceae*, specifically *P. ruminicola* 23, during primary colonisation, before decreasing during secondary colonisation (Figure 3). This suggests a role for QS as a driver for the switch between sub-microbiomes of primary and secondary colonisation [6].

When considering consequences of QS on the production of livestock, differing levels of QS, based on metagenomic techniques employed by Xie et al [25], has been linked to different animal phenotypes including residual feed intake (RFI). RFI is defined as “The difference between the actual and expected feed intake of an animal based on its body weight and growth rate over a specified period.” [51]. Therefore, an animal with high efficiency of feed conversion is expected to have a low RFI, with a high RFI indicating an inefficient animal. QS in the rumen was significantly higher in cattle with high RFI, specifically three genes, EC 4.1.1.15, EC 4.2.2.2 and EC 6.2.1.32, known to be regulated via QS systems were enriched in high RFI animals [25]. These enriched genes encoded the following enzymes; glutamate decarboxylase [52], pectate lyase [53] and acetate-CoA ligase [54]. These lower efficiency animals (high RFI) also had a significantly higher predicted methane production (p = 0.04) of 533 g/d when compared to the low RFI animals estimated to produce 506 g/d [25]. (A mathematical model was employed to predict methane production by the equation: CH4 (MJ/d) = 3.23 (±1.12)+0.81 (±0.086)×dry matter intake (kg/d) [55]). Animals with high RFI also had 6 methane metabolism genes, EC 1.5.98.1, EC 1.5.98.2, EC 2.1.1.248, EC 2.1.1.86, EC 2.1.1.90 and EC 2.3.1.101, enriched in their rumen microbiome [25]. In contrast, the more efficient animals, with lower RFI, were found to have lower levels of methanogenesis as well as lower abundances of *Methanosphaera* and *Methanobrevibacter ruminantium* [25]. Twenty-two bacterial species were identified as contributors to the differences observed in feed efficiency between the animals. These species were associated with both QS and carbohydrate metabolism capacity and included taxa belonging to the family *Lachnospiraceae* and included *Ruminococcus torques*, *Blautia obeum*, *Blautia producta*, *Blautia schinkii*, *Blautia wexlerae*, *Dorea longicatena*, *Enterocloster clostridioformis* (*Clostridium clostridioforme*), *Clostridium symbiosum*, *Marvinbryantia formatexigens*, and *Lachnospiraceae bacterium* [25]. Nonetheless, despite studies now showing the presence of QS in the rumen microbiome and correlating such activity to animal phenotype, the influence of QS on subsequent gene regulation is still unclear and requires further research.

## CONCLUSION

Quorum sensing mechanisms play a crucial role in the rumen microbiome, impacting competition, symbiosis, and functional dynamics. However, studying QS in complex microbiomes, like the rumen, faces challenges due to the limitations associated with existing methodologies. To advance our understanding, we need comprehensive approaches like comparative genomics, refined anaerobic cultivation, and co-culture investigations. Despite significant strides in our knowledge of the rumen microbiome and its functionality, our comprehension of QS and its specific role within this niche remains understudied. Understanding these signalling pathways could empower us to manipulate the microbiome for animal and productivity benefits. This knowledge could contribute to developing direct-fed microbials, promoting sustainability, animal health, and enhanced productivity. Beyond reducing methane emissions, this approach aligns with planetary health goals by improving animal performance and meat quality. In summary, unravelling QS intricacies in the rumen microbiome is vital for advancing animal health and overall agricultural sustainability.
